# Supplementary figures and images for: Optimization of a deep mutational scanning workflow to improve quantification of mutation effects on protein–protein interactions
Source: BMC Genomics. 2024 Jun 24;25:630. doi: 10.1186/s12864-024-10524-7 (PMC11194945; doi:10.1186/s12864-024-10524-7)

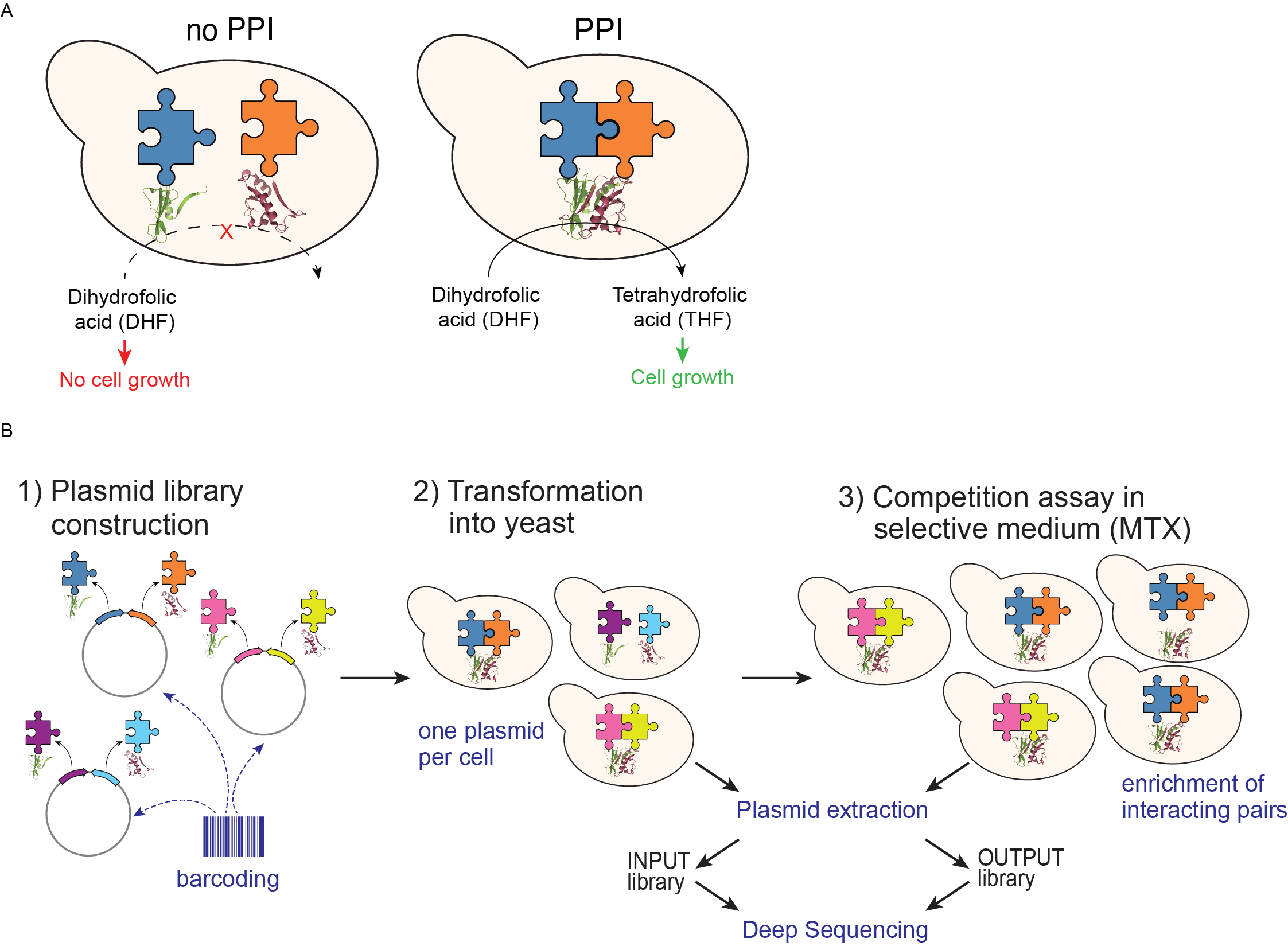

Supplement: Supplementary file 1 — Supplementary Material 1. [file 12864_2024_10524_MOESM1_ESM.png]

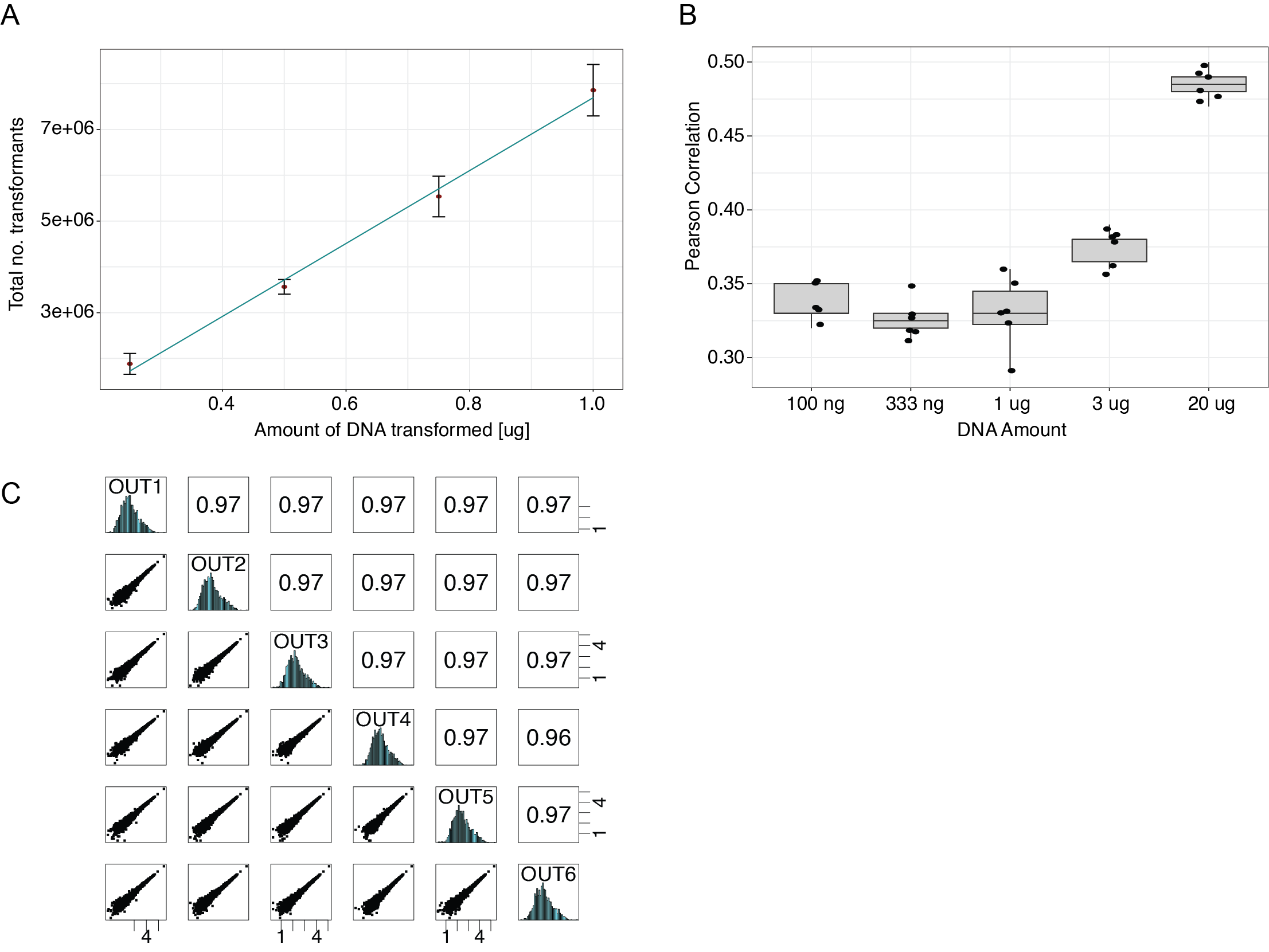

Supplement: Supplementary file 14 — Supplementary Material 14. [file 12864_2024_10524_MOESM14_ESM.png]

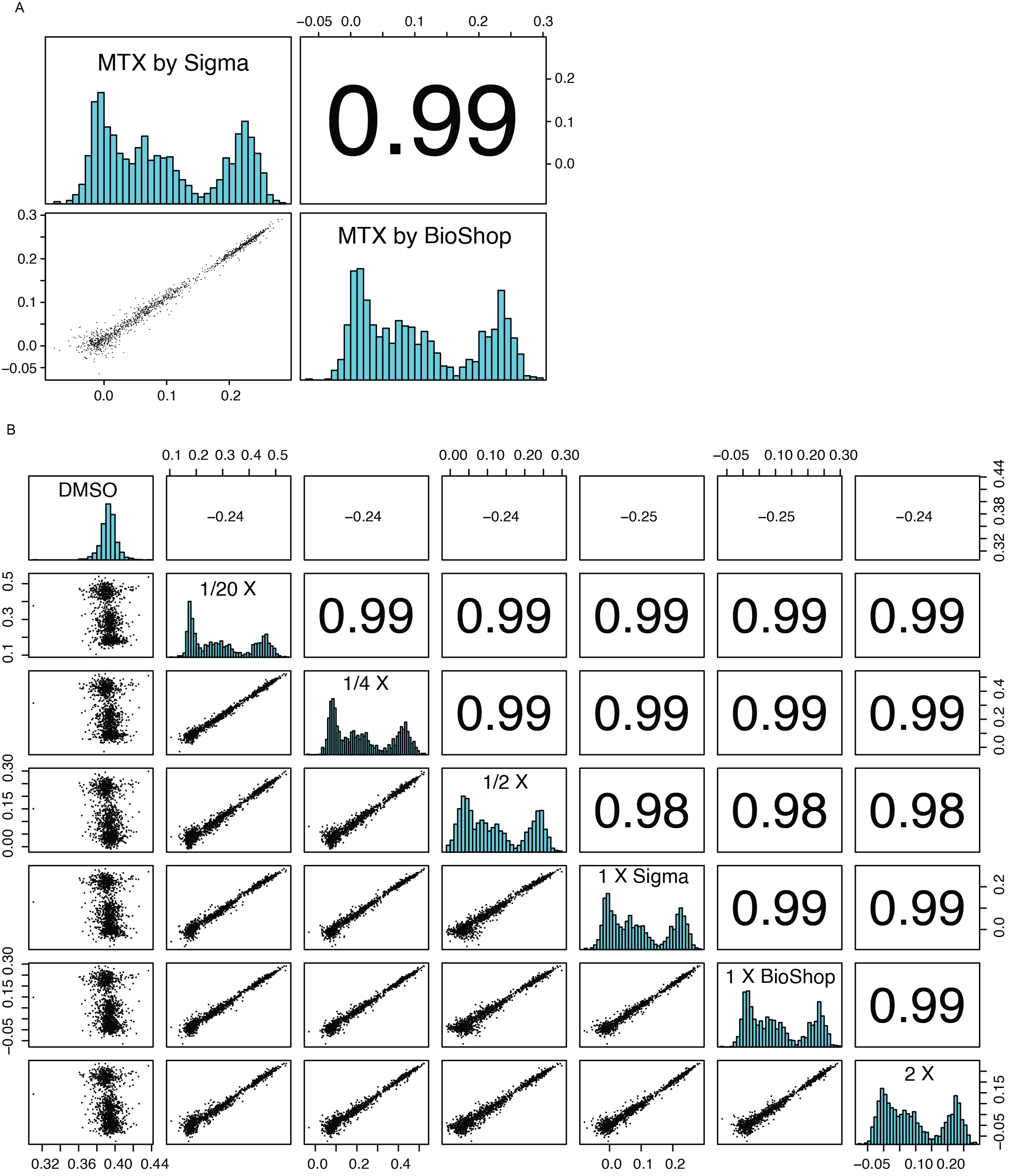

Supplement: Supplementary file 15 — Supplementary Material 15. [file 12864_2024_10524_MOESM15_ESM.png]

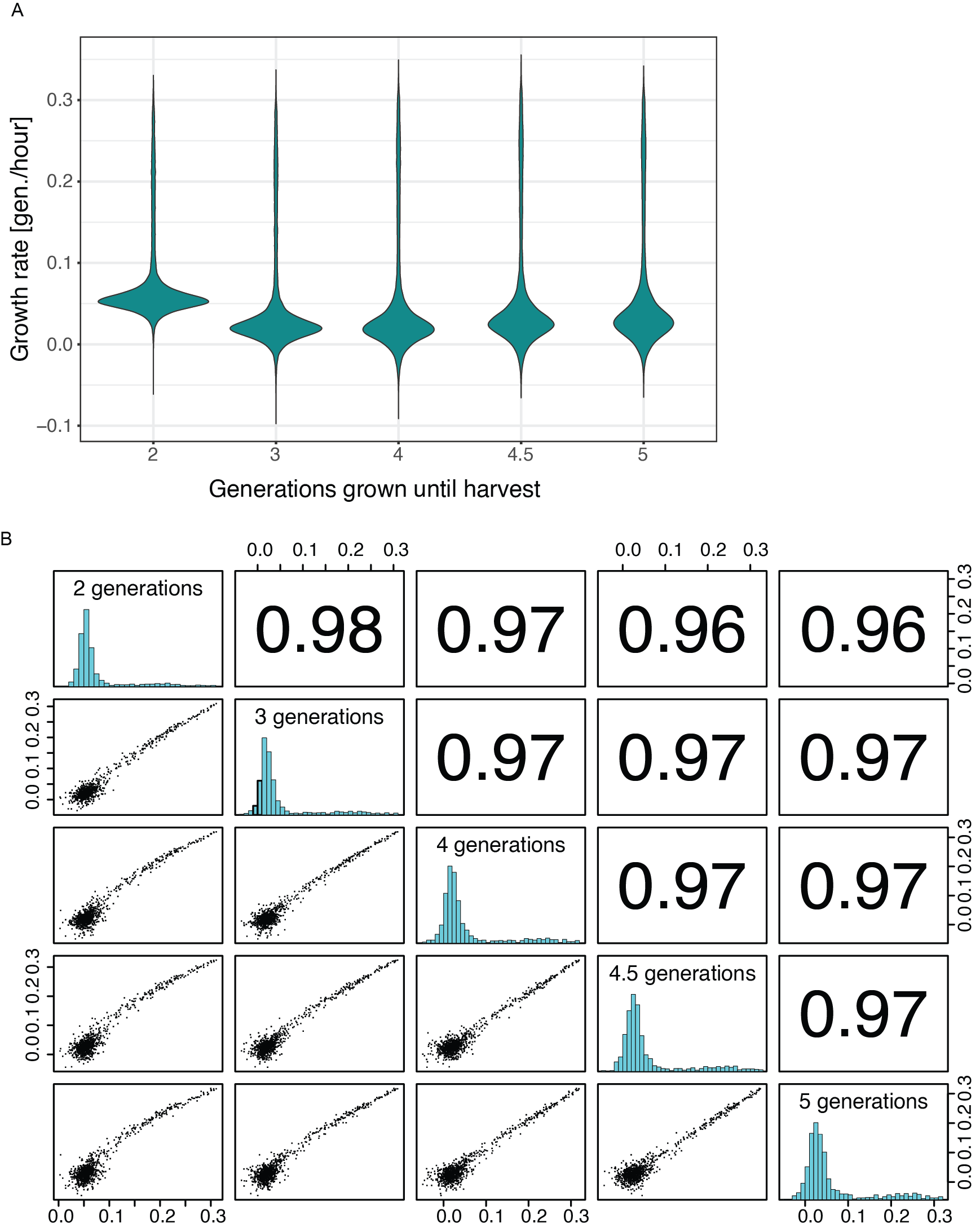

Supplement: Supplementary file 16 — Supplementary Material 16. [file 12864_2024_10524_MOESM16_ESM.png]
